# Modeling temporal sequences of cognitive state changes based on a combination of EEG-engagement, EEG-workload, and heart rate metrics

**DOI:** 10.3389/fnins.2014.00342

**Published:** 2014-11-05

**Authors:** Maja Stikic, Chris Berka, Daniel J. Levendowski, Roberto F. Rubio, Veasna Tan, Stephanie Korszen, Douglas Barba, David Wurzer

**Affiliations:** ^1^Advanced Brain Monitoring Inc.Carlsbad, CA, USA; ^2^Center for Performance Psychology, National UniversityCarlsbad, CA, USA

**Keywords:** unsupervised learning, self-organizing map, cognitive state, electroencephalography (EEG), electrocardiography (ECG)

## Abstract

The objective of this study was to investigate the feasibility of physiological metrics such as ECG-derived heart rate and EEG-derived cognitive workload and engagement as potential predictors of performance on different training tasks. An unsupervised approach based on self-organizing neural network (NN) was utilized to model cognitive state changes over time. The feature vector comprised EEG-engagement, EEG-workload, and heart rate metrics, all self-normalized to account for individual differences. During the competitive training process, a linear topology was developed where the feature vectors similar to each other activated the same NN nodes. The NN model was trained and auto-validated on combat marksmanship training data from 51 participants that were required to make “deadly force decisions” in challenging combat scenarios. The trained NN model was cross validated using 10-fold cross-validation. It was also validated on a golf study in which additional 22 participants were asked to complete 10 sessions of 10 putts each. Temporal sequences of the activated nodes for both studies followed the same pattern of changes, demonstrating the generalization capabilities of the approach. Most node transition changes were local, but important events typically caused significant changes in the physiological metrics, as evidenced by larger state changes. This was investigated by calculating a transition score as the sum of subsequent state transitions between the activated NN nodes. Correlation analysis demonstrated statistically significant correlations between the transition scores and subjects' performances in both studies. This paper explored the hypothesis that temporal sequences of physiological changes comprise the discriminative patterns for performance prediction. These physiological markers could be utilized in future training improvement systems (e.g., through neurofeedback), and applied across a variety of training environments.

## Introduction

Passive Brain Computer Interface (BCI) utilizes spontaneously occurring brain signals to implicitly infer information about the cognitive or affective state of the user. Zander and Kothe (2011) presented a wide range of scenarios for the general population, i.e., healthy users that do not have difficulties to move or speak. This opens a number of new challenges, as it is difficult to compete with the standard communication channels such as speech and interaction devices (e.g., a mouse or keyboard) available to healthy users (Brouwer et al., 2013). However, passive BCIs provide an implicit input modality that carries information about the actual user cognitive or affective state that is not intentionally sent by the user. This offers numerous opportunities for adaptive human-computer interaction techniques that would adjust to the ongoing user's mental state (Cutrell and Tan, 2008; Zander et al., 2008; George and Lecuyer, 2010). Novel applications for passive BCI technology in domains such as entertainment, gaming, marketing, and industry continue to emerge (van Erp et al., 2012), e.g., implicit tagging of multimedia content, different avatar's appearances reflecting the gamers' mental state, adaptive automation in driving environments depending on the driver's workload, etc. Insight into the cognition and internal mental state of the user provides valuable information for detection of emergency due to the lack of concentration, drowsiness, or too high mental workload in safety-critical applications, such as driving or security surveillance (Zander and Kothe, 2011). Furthermore, training and education could also benefit from a more in-depth understanding of cognitive and affective states during learning with technology designed for cognitive enhancement and neurofeedback (Berka et al., 2010; van Erp et al., 2012).

A large number of previous studies in the context of training and education focused on error detection and performance prediction based on brain activity (Gehring et al., 1993; Falkenstein et al., 2000; Ferrez and Millan, 2008; Lehne et al., 2009; Stikic et al., 2011). This line of research has shown great promise in providing the means to increase the pace of skill learning, which is typically characterized as a three stage process (Fitts and Posner, 1967). In the initial cognitive stage, new knowledge is assembled to understand the skill to be learned (i.e., what to do) by effortful processing of perceptual cues. In the next, the associative stage, sensory processing of relevant stimuli is refined through practicing (i.e., how to do it) until reaching the third, the autonomous stage, which is characterized by automated execution of the task with minimal conscious mental effort. A growing body of research has been investigating physiological patterns associated with skill acquisition, mostly in sport environments that require complex visuomotor coordination, ranging from archery (Salazar et al., 1990; Landers et al., 1994), over marksmanship (Haufler et al., 2000; Hillman et al., 2000; Kerick et al., 2001; Deeny et al., 2003), to golf (Crews and Lander, 1993; Babiloni et al., 2008). These tasks are also characterized by a motionless preparatory period (“pre shot routine”) before the skilled movement occurs. Since there are minimal movement artifacts during this preparatory period, EEG recordings in such settings are feasible. Gevins et al. (1999) suggested that EEG is more effective than other neuroimaging techniques in providing dynamic measures of skill acquisition due to the high temporal resolution, its online monitoring ability, and the potential for real-time feedback based on cognition, attention, and arousal.

All above mentioned skill acquisition studies focused on the analysis of low-level EEG features such as Power Spectral Densities (PSDs) grouped into the standard bandwidths, or EEG coherence analysis. Salazar et al. (1990) found alpha power in the left hemisphere to be dominant during the aiming period in archery. Haufler et al. (2000) compared the EEG of novices and expert shooters, finding that the experts exhibited less activation than the novice shooters during the aiming period, with a pronounced difference in the left central-temporal-parietal area. Crews and Lander (1993) suggested a decrease in left hemisphere, motor cortex activity as golf players prepared to putt. Babiloni et al. (2008) found that stronger event-related desynchronization (ERD) in high frequency alpha power (10–12 Hz) over the frontal midline (Fz and Cz) and right primary sensorimotor cortex (C4) is correlated with successful golf putts. The results of a study conducted by Deeny et al. (2003) revealed that, compared to lesser skilled shooters, experts engaged in less cortico-cortical communication, particularly between left temporal association and motor control regions, which implies decreased involvement of cognition with motor processes.

Changes in heart rate (i.e., the number of heartbeats per unit of time) have also been analyzed in relation to expert performance, and they are believed to reflect the focusing of attention and the skill related aspects of sensory-motor preparation for performance. The pre-shot period is typically characterized by heart rate deceleration (Landers et al., 1994; Kontinnen et al., 1998; Tremayne and Barry, 2001).

Building upon these findings, Berka et al. (2010) went one step further by developing a device called the Adaptive Peak Performance Trainer (APPT) that provides continuous psychophysiological monitoring and feedback (i.e., visual, auditory, and haptic) to the trainee in real-time. The device was based on a unique identified Pre-Shot Peak Performance (PSPP) expert profile in three different tasks (i.e., marksmanship, archery, and golf), which was characterized by an increase in midline theta and left temporal-parietal alpha EEG frequency bands and a local deceleration of heart rate. Skill learning was accelerated significantly when novices were trained with the APPT compared to controls, suggesting its feasibility as a training aid.

None of these past studies incorporated higher level cognitive state metrics such as EEG-engagement and EEG-workload that could potentially also relate to an individual's skill development. These metrics could contribute to better generalizability across different tasks compared to the low level EEG based features. Engagement reflects the allocation of processing resources (Anderson, 2004) including the cognitive processes associated with decision making, such as information gathering, visual scanning, audio processing, and selectively sustaining concentrated attention on one aspect of the environment while ignoring other distractions. Workload is typically defined as the amount of mental or physical resources required to perform a particular task (Son and Park, 2011). Seidler et al. (2012) have shown that individual differences in spatial working memory capacity predict the rate of motor skill learning. Bainbridge (1989) argued that workload is reduced with the development of a certain skill. However, Brouwer et al. (2014) did not find physiological variables to indicate decreasing effort associated with learning. EEG-based neural indices of cognition that reflect levels of alertness, selective attention, and working memory have been identified in previous studies (Hillyard et al., 1973; Gevins et al., 1998; Mikulka et al., 2002). However, these indices have only started to be explored in skill acquisition and performance prediction studies. Moreover, the neurophysiological data are typically analyzed during the pre-shooting period only, neglecting the changes over the entire process that include, for example, observation of the dynamic environment, evaluating possible threats, and eliminating enemies in the marksmanship scenarios. Lastly, the previous studies did not account for the individual variability in the EEG data, and many of them lacked evaluation of the generalizability of the proposed approaches across different users and tasks.

Performance prediction algorithms are based on either supervised or unsupervised machine learning methods. The main difference between these two types of methods is whether they utilize labeled data (supervised learning) or unlabeled data (unsupervised learning). Many of the supervised approaches are not based on EEG data, but on kinematical models, e.g., using computer vision. Couceiro et al. (2013) compared different classifiers (linear and quadratic discriminant analysis, Support Vector Machines—SVM, and naive Bayes) in their golf study. The experimental results showed the superiority of the SVM classifier, however, a large individual variability in the data was present due to the different game styles of each player. Jensen et al. (2012) evaluated AdaBoost, Fisher LDA, k-Nearest Neighbor (kNN), naive Bayes, and SVM classifiers in a study of kinematic golf putt data classification based on the inertial sensor. Nagashima et al. (2009) assessed marksmanship performance by sensor-based breath and trigger control measures that were fitted with a logistic regression model. Muangjaroen and Wongsawat (2012) developed an EEG-based real-time index for predicting putt success based on high alpha power in the C4 sensor site, theta power in Fz, and both high alpha and theta power in Pz. An impeding bottleneck of the supervised learning methods is the substantial amounts of labeled training data required to train a classifier. Labeling of data is typically a labor-intensive, time-consuming, and error-prone process that scales poorly to the large number of users and skill learning sessions necessary to achieve the required level of expertise. This limits the applicability of the supervised learning approaches in challenging skill acquisition settings where fine grained labels of all relevant session's periods would need to be precisely obtained. Another line of research avoids the labeling efforts by unsupervised discovery of structure in the data. However, there are only a few studies where unsupervised approaches were applied for performance prediction, such as data fusion of EEG and ECG signals through Self-Organizing Maps (SOM) (Bandeira et al., 1999). In this approach, shooting data were classified by majority voting of the labeled neurons of the trained SOM. However, this study focused on the data fusion and analyzed only low-level EEG features. The study included a single constrained shooting scenario with only seven subjects. Labeling of the neurons consisted of finding the “winning” neuron in a SOM for each feature vector in the dataset, and assigning the feature vector's label to the neuron's label. Thus, this approach also required labeled training data. The activated NN nodes represented the single subject's data, and did not include similar performance data. Data fusion slightly improved performance of the classifier, but they did not conduct cross-validation of the algorithm on an independent dataset or made any effort to determine whether the model would generalize across tasks.

In this paper, we present an alternative approach toward performance estimation in dynamic environments requiring rapid decisions. We focus on the two tasks that were often analyzed in the past: combat marksmanship (e.g., Haufler et al., 2000; Deeny et al., 2003) and golf training (e.g., Crews and Lander, 1993; Babiloni et al., 2008). These two tasks both require complex visuomotor coordination, mental and sensory-motor preparation, focused attention, and concentration. None of the previous studies in the literature explored the potential similarities of the neurophysiological performance predictors on these tasks. This novel approach is based on a combination of the two types of higher level EEG-based cognitive state measures associated with decision-making, alertness, attention, and working memory: engagement (Johnson et al., 2011) and workload (Berka et al., 2007). Furthermore, heart rate is also included in the analysis, as it adds the dimensions of stress, anxiety, frustration, and arousal experienced by a shooter (Berka et al., 2010). EEG-engagement reflects information-gathering, visual processing, and allocation of attention, while EEG-workload is associated with increased working memory load during problem solving, integration of information, and analytical reasoning (Berka et al., 2007). The EEG-based metrics are used as an indicator of how well a shooter is processing information, as well as an indicator of the focused and relaxed mental state that is a prerequisite for good shooting performance (Raphael et al., 2009). We tested the following hypotheses: (1) temporal sequences of EEG-based engagement and workload and ECG-based heart rate comprise discriminative patterns related to performance on the combat marksmanship task, (2) the discovered patterns are good indicators of performance on the golf training task, and (3) individual differences in the neurophysiological metrics could be overcome by data normalization. In order to overcome the main drawback of the supervised approaches that require large amounts of labeled training data, we explored the potential of the unlabeled data by applying an unsupervised approach. The approach is based on the one dimensional self-organizing NN inspired by work of Stevens et al. (2009, 2013). However, we extended their algorithm by combining all three metrics together (i.e., EEG-engagement, EEG-workload, and heart rate) into a single NN. Multiple neurophysiological metrics could facilitate developing a deeper understanding of skill acquisition, as they have different functional properties reflecting various aspects of the learning process. Furthermore, we incorporated the temporal modeling of these metrics on a fine-grained scale by including 3 consecutive seconds into a feature vector. All metrics were self-normalized to account for the individual differences inherent in the physiological data. The NN model was trained and auto-validated on the combat marksmanship training data. The trained model was then cross-validated in two different ways. First, we utilized 10-fold cross-validation (Kohavi, 1995) to evaluate generalization capabilities of the approach across different subjects. Second, we also evaluated the generalization capabilities of the model by cross-validating it on the golf-training data. Correlation analysis of the temporal cognitive state changes was performed to investigate if they comprise the discriminative patterns for performance prediction that could ultimately allow for faster learning of different skills through neurofeedback. Lastly, the model was compared to the linear regression model on the same set of variables to investigate whether the utilized variables or their temporal modeling with the NN approach contributed to the results.

## Materials and methods

Two studies were conducted: combat marksmanship training on the military simulator, and golf putting training at the indoor golf facility. In this section, we describe the protocols of both studies, and we detail data recording setup, signal processing, and algorithm development procedures.

### Participants

For the combat marksmanship study, a total of 51 participants (22 females and 29 males, mean age 27.4 ± 6.7 years) were recruited from local colleges and newspaper/online advertisements. All participants had normal or corrected-to-normal vision. No participants that had undergone formal marksmanship training were admitted to the study. Informed consent was obtained from all participants in accordance with the guidelines and approval of the Biomedical Research of America IRB.

A total of 22 subjects were recruited for the golf training study using the National University Golf Academy's (NUGA) IRB approved protocol. The participants were split into two groups: experienced players and novices, based on their previous experience. Each group had 11 participants and in each group 45% of the population was male. Average overall age was 33.9 ± 11.1.

No study procedures were conducted until the consent form was fully explained and the consent form was signed. There was no overlap between the participants in these two studies. All participants in both studies were right-handed.

### Study protocol

The study protocols were comprised of screening, baseline, and the experimental session.

#### Screening procedure

The eligibility screening criteria aimed to cover anything known to effectively alter the EEG signals, so those subjects with general health problems such as psychiatric, neurological, behavioral, attention, or sleep disorders, as well as any pulmonary or eating disorder, diabetes, high blood pressure, or history of stroke were excluded from the studies. Those who reported using pain medications regularly, stimulants such as amphetamine, illicit drugs, or those who consumed excessive alcohol or tobacco on a daily basis were also excluded. Exclusion criteria also included head injuries within the past 5 years and pregnancy. Eight subjects failed this initial screener. Participants who passed the screener were scheduled for the baseline session. Participants were asked to ensure they got a full night of sleep (between 7.5 and 9 h) in the night leading up to their study appointments. Participants were also requested to refrain from drinking alcohol at least 24 h before each study visit, and abstain from caffeine on the day of the study and nicotine 1 h before the start of the study visit.

#### Baseline session

In both studies, the participants completed a 15 min baseline (BL) using the Alertness Memory Profiler (AMP). The AMP was developed by Advanced Brain Monitoring Inc. (ABM) to integrate EEG, ECG, and performance measures in an easy-to-administer platform designed for quantitative assessment of neurocognitive functions, including alertness, attention, learning, and memory. The AMP uses a multivariate approach that allows simultaneous acquisition and analysis of data.

**Figure 1:**
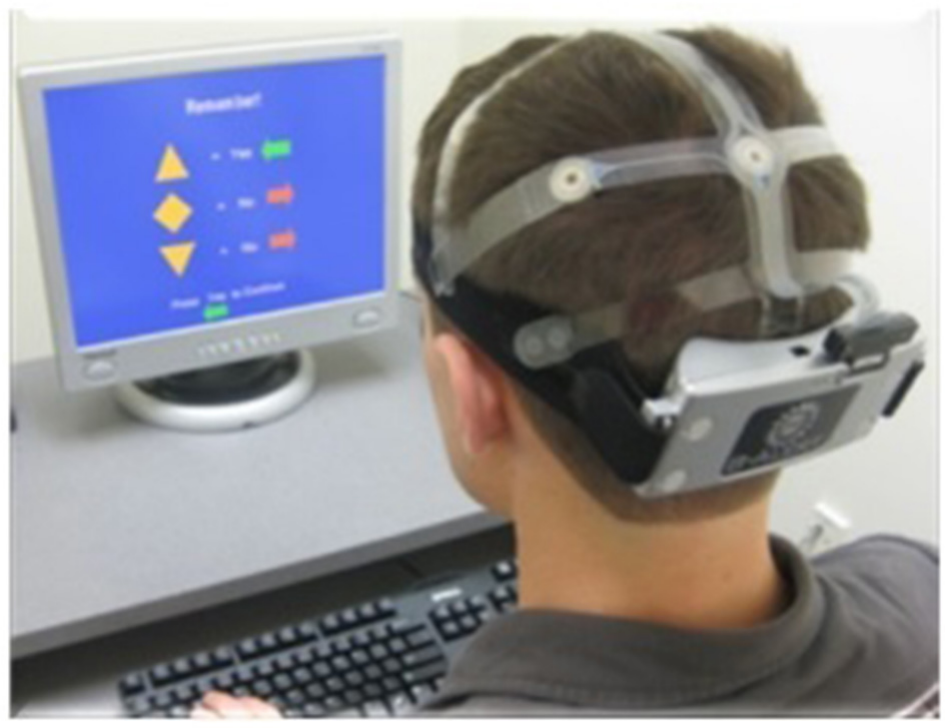
**A subject wearing the wireless EEG sensor headset while performing the 3CVT task**.

The baseline session comprised a Visual Passive Vigilance Task (VPVT), an Auditory Passive Vigilance Task (APVT), and a 3-Choice Active Vigilance Task (3CVT). During the 5 min of VPVT and APVT, subjects were required to press the space bar on the keyboard every 2 s. Subjects were prompted to maintain the 2 s time intervals by a 10 cm diameter red circle that appeared in the center of the monitor during the VPVT, or by an audio tone during the APVT. The 3CVT (Figure 1) is a sustained attention task that requires subjects to discriminate one primary target (presented 70% of the time) from two secondary non-target geometric shapes that were presented for 0.2 s and randomly interspersed over the test period. It challenges the ability to sustain attention by increasing the inter-stimulus interval (ISI) from 1.5 s up to 10 s at the end of the task. Participants were instructed to respond as quickly and as accurately as possible to each stimulus presented by pressing the left arrow to indicate target stimuli, and the right arrow to indicate non-target stimuli. A brief training period was provided prior to the start of the testing period to minimize practice effects for the 3CVT. The training period lasted until a certain number of correct responses to both targets and non-targets was reached (2 targets and 2 non-targets). The applied criterion was the same for all participants in both studies. On average, training period lasted 30 s.

#### Combat marksmanship training experiment

**Figure 2:**
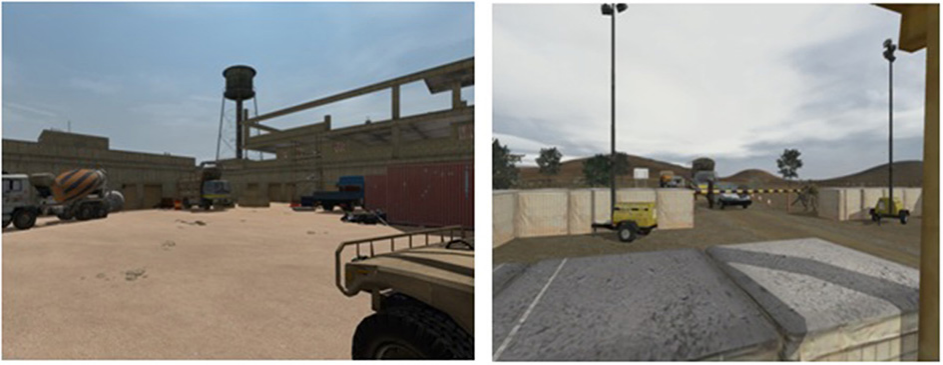
**Combat marksmanship scenario scenes**.

The military training platform Virtual Battle Space 2 (VBS2), Tactical Warfare Simulation running on the Real Virtuality 2 simulation engine and developed by Bohemia Interactive, was used to create the combat marksmanship training scenarios. The VBS2 platform was designed in close cooperation with the United States Marine Corps and Australian Defence Force, and it has been used as a training battlefield simulation system by federal, state, and local government agencies worldwide for tactical training and mission rehearsal in military organizations. It is an interactive, 3D training system that allows users to construct specific missions based on their individual needs. In collaboration with Laser Shot Inc. and a subject-matter expert (SME) from Washington State University, we developed five custom combat scenarios using VBS2. The scenarios (Figure 2) had realistic settings and contexts in which participants (acting as soldiers) were required to make deadly force decisions. In order to mimic reality as closely as possible, the room was equipped with life-size projection of threats, stereo sound delivered via earbuds, and other paraphernalia found in the battlefield environment. The participant used a demilitarized “airsoft” replica of an M4 rifle that interacted with the platform using a wireless laser-based training system from Laser Shot Inc. The M4 was mounted with a holographic weapon sight, or red dot scope, commonly used in a combat environment for quick target acquisition. Sandbags were provided to support the weight of the weapon, to both simulate combat firing procedures and investigate the effect of muscle fatigue on performance. The weapon also had a CO_2_ gas recoil system that simulated the kickback of the weapon.

Participants were initially given marksmanship instructions and requested to undergo a set of training tasks. Training addressed the fundamentals of marksmanship (aiming, breath control, trigger control, etc.) and the rules of engagement applicable in the testing scenarios. During the three training scenarios, the participants practiced firing the M4: (1) at close-range static targets, (2) at targets of various distances, and (3) at targets that were randomly displayed among non-targets. Five testing scenarios were administered in a randomized order for each subject. Each scenario was set in a unique environment that replicated typical fire-fighting situations for soldiers (e.g., Afghanistan compound, checkpoint, and market; Improvised Explosive Device (IED) compound, or urban alley). In order to avoid excessive fatigue in participants, the scenarios were designed from a fixed point of view and each scenario lasted only 3–4 min, on average. Throughout the scenarios, a mixture of enemy and friendly units (both stationary and moving) appeared at varying distances. Two scenarios were enemy-heavy in which 90 and 76% of the units were enemies, two were friendly-heavy in which 76 and 60% of the units were friendly, and one had about equal mix of enemy and friendly units (47% enemy units and 53% friendly units). Participants were instructed to evaluate threats and eliminate all enemy units as quickly as possible.

To enable detailed event tracking, an event logging and synchronization platform was utilized as an essential piece for the study testbed. An External Sync Unit (ESU) was used to synchronize the physiological signals with the relevant events in the scenarios (such as friend/enemy became visible, enemy fired, etc.), as well as user responses (e.g., rifle shots, hit friend/enemy, etc.). Synchronization in the Windows environment is dependant on the windows task scheduler and cannot guarantee an upper bound for user level tasks, while the ESU is a general purpose data integration platform that can synchronize multi-source digital data (serial and/or parallel port protocols) with physiological signals to millisecond-level precision.

#### Golf training experiment

**Figure 3:**
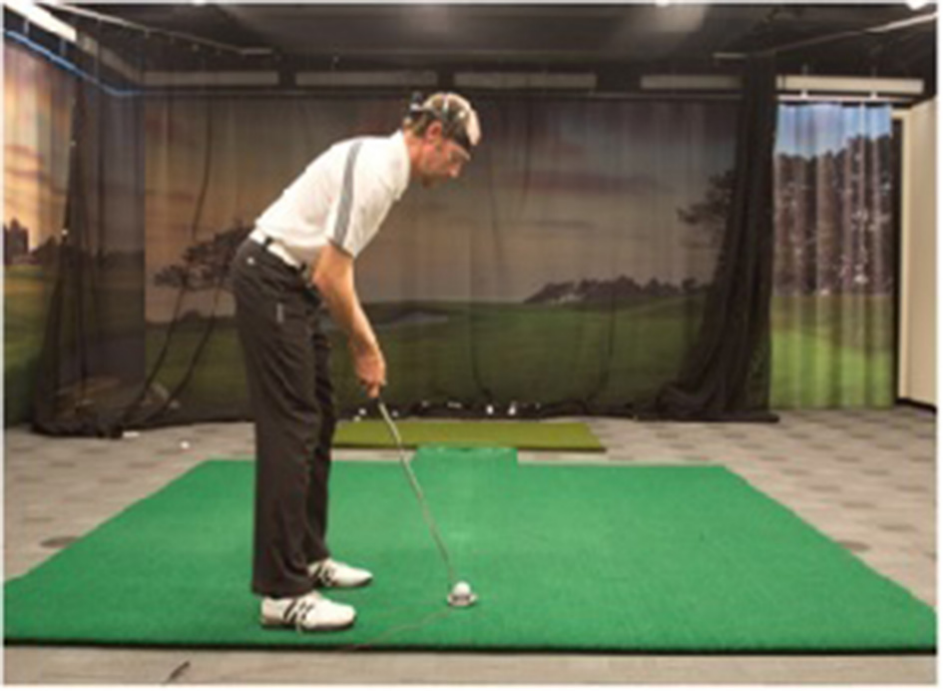
**NUGA's golf facility**.

**Figure 4:**
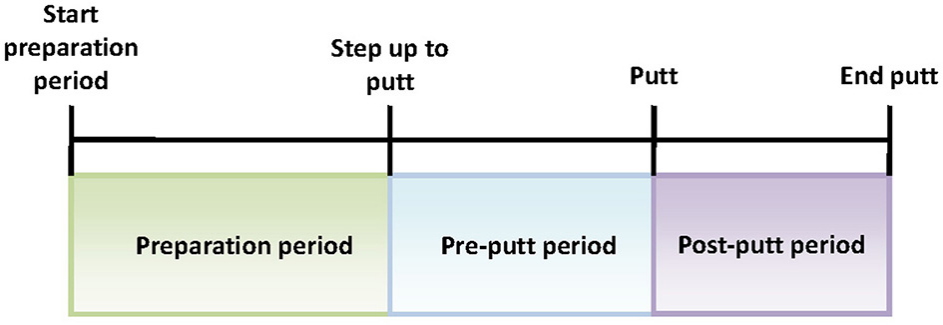
**Putt periods of interest**.

The golf training experiment was designed in close cooperation with SMEs from NUGA. The participants were asked to come to the indoor golf facility at the NUGA's premises. Upon arrival, they were asked to complete 10 sessions of 10 putts each wearing the wireless EEG headset (Figure 3). The average session duration was 5.5 min. It was decided that 100 putts from each subject would be required for the EEG data analysis. To avoid possible fatigue, those 100 putts were broken down into 10 sessions. All sessions were administered within the same day, with 3 min breaks between the sessions. The putting distance was far enough (10 feet) to be challenging for both experienced players and novices. Each putt comprised a series of steps including: (1) preparation period—the participants were asked to stand a few feet behind the ball before stepping up to the ball; (2) step up to putt—when the subjects were ready, they would walk up to the ball and get into their putting stance; (3) start putt—when the putter made contact with the ball; and (4) end putt—motion of the ball was stopped or off the green. All relevant events were manually marked and logged during the real-time EEG acquisition and for each putt the three signal sequences of interest were analyzed: preparation period, pre-putt period, and post-putt period (shown in Figure 4).

### Data recording and signal processing

EEG and ECG data were collected using the wireless EEG sensor headset (Figure 1) developed by ABM (Berka et al., 2004). For the combat marksmanship study, nine Ag/AgCl EEG electrodes were located at F3, Fz, F4, C3, Cz, C4, P3, POz, P4, according to the international 10–20 system. For the golf study, 20 EEG channels were utilized: Fp1, Fp2, Fz, F3, F4, F7, F8, T3, T4, T5, T6, Cz, C3, C4, Pz, P3, P4, O1, O2, and POz sites. The EEG channels were referenced to linked reference electrodes located behind each ear on the mastoid bone. Even though different montage was used in these two studies, only the data from the EEG channels that are necessary for calculating EEG-based engagement and workload metrics were analyzed: Fz, F3, Cz, C3, C4, and POz. The most discriminative variables for both models (i.e., EEG-engagement and EEG-workload) were selected in the previous study (Berka et al., 2007) using stepwise regression. ECG was recorded with electrodes placed on the clavicle and opposite lower rib. All data was sampled at 256 Hz and transferred in real-time via Bluetooth link to a nearby host computer where the proprietary data acquisition software stored the data onto the disk.

The EEG signals were filtered with a band-pass filter (0.5–65 Hz) before the analog-to-digital conversion. To remove environmental artifacts from the power network, notch filters at 50, 60, 100, and 120 Hz were applied. The decontamination algorithm (Berka et al., 2004) detected and removed any sudden changes in amplitude, i.e., artifacts in the time-domain EEG signal, including spikes caused by tapping or bumping of the sensors, amplifier saturation, and excursions. Eye blinks were identified and decontaminated by wavelet transform based upon an algorithm presented in Berka et al. (2007). The wavelet decomposition coefficients for differential Fz-POz and Cz-POz EEG channel derivations in the exponential 0–2, 2–4, 4–8, 8–16, 16–32, 32–64, and 64–128 Hz bands were calculated by applying the Coiflet order-1 wavelet filter (Wei, 1998). These coefficients were further utilized in the discriminant function analysis to detect the eye blink regions. Decontamination of eye blinks was accomplished by computing mean wavelet coefficients from the nearby non-contaminated regions and replacing the contaminated data points. The EEG signal was then reconstructed by wavelet composition. From the filtered and decontaminated EEG signal, the log-transformed absolute power spectral densities (PSD) were calculated for each 1 s epoch of data by applying Fast Fourier Transformation (FFT). Relative PSD values were derived by subtracting the PSD for each 1 Hz bin from the total PSD power in the range of 1–40 Hz. Lastly, excessive muscle activity (EMG) was detected by identifying epochs in which (a) PSD bins from 35 to 40 Hz were above a certain threshold and (b) the square root of the PSD bins' sum from 70 to 128 Hz was above a defined cut-off value. The threshold values were customized empirically by visual inspection of the large EEG data cohort contaminated with EMG during the EEG sensor development. Epochs with detected EMG artifacts were discarded from further analysis. On average, 21.9 and 23.4% of the epochs per session were contaminated with artifacts in the combat marksmanship and golf study, respectively. There were no significant differences in the number of detected artifacts for different combat marksmanship scenarios (19.9, 20.6, 21.2, 23.4, and 24.2%).

The ECG signal was filtered with the band-pass 4th order IIR filter (15–35 Hz). This improved the contrast between the QRS complex and the T wave, and minimized double peak detection, leading to the more robust detection of peaks. The filtered signal was processed by a real-time algorithm that computed sample-by-sample running mean and standard deviation to derive a threshold for peak detection. The inter-beat (R-R) interval was determined as the number of seconds between the current and the previous peak. Heart rate was estimated as a number of beats per minute, i.e., 60/(R-R interval), and logged in real-time. The algorithm assessed the quality of detected beats by monitoring the standard deviation of up to 6 consecutive beats.

### EEG-based engagement and workload metrics

In order to explore the applicability of alertness quantification in performance estimation, we incorporated the B-Alert® model (Johnson et al., 2011) into the analysis. It is an individualized model that classifies a subject's cognitive state into different levels of alertness: distraction/relaxed wakefulness, low engagement, or high engagement. It utilizes the absolute and relative PSD values from the midline Fz-POz and Cz-POz derivations during VPVT, APVT, and 3CVT BL data to derive coefficients for a discriminant function that generates classification probabilities for each 1 s epoch. During the model training procedure, APVT, VPVT, and 3CVT represent distraction/relaxed wakefulness, low engagement, and high engagement, respectively. The quality of the individual models were assessed by auto-validation on these three tasks (APVT, VPVT, and 3CVT), and for all subjects the majority of the epochs were classifed into the expected class. This model has been validated in a number of previous studies across a range of applications, such as sleep deprivation (Westbrook et al., 2004), team collaboration (Stevens et al., 2009), and emergent leadership (Waldman et al., 2013). The high engagement output posterior probabilities of the model were added to the feature vector for further analysis.

The EEG-based workload model (Berka et al., 2007) was utilized to extract the subject's cognitive workload levels on a second-by-second basis. This is a general model that was trained on the EEG data from a large population performing the Forward Digit Span (FDS) and Backward Digit Span (BDS) tasks. During these two tasks, the subjects sit still in front of a computer screen and memorize sequences of 2 up to 9 digits that are shown on the computer screen as a series of single digits of increasing lengths, followed by an empty box prompting the participant to reproduce the sequence by typing in the memorized digits in the presented order (FDS) or the reverse order (BDS). For both FDS and BDS, the task difficulty was manipulated by increasing the number of digits at each level. The model utilizes the absolute and relative PSDs from the differential EEG derivations (C3-C4, Cz-POz, F3-Cz, Fz-C3, and Fz-POz) into a 2-class linear Discriminant Function Analysis classifier (i.e., low workload and high workload). The present study employed the posterior probability of the high workload class in order to identify a continuous measure of EEG-workload and analyze its temporal changes during skill training.

It has been shown in the previous studies (Stevens et al., 2013) that EEG-engagement and EEG-workload metrics have different functional properties in response to different tasks and they are poorly correlated with one another. Correlation between EEG-engagement and EEG-workload was *R* = −0.24 ± 0.21 with an *R*^2^ of 0.11 ± 0.11 across the marksmanship training sessions. Correlation between EEG-engagement and EEG-workload across the golf training sessions was *R* = −0.11 ± 0.22 with an *R*^2^ of 0.06 ± 0.11. Correlation between these two metrics in Stevens et al. (2013) was in the same range *R* = −0.19 ± 0.24 (*R*^2^ = 0.09 ± 0.05).

### Performance metrics

In both the combat marksmanship and golf training studies, the subjects' performance scores were calculated. For the combat marksmanship data, the following metrics were included in the analysis: percent of enemy hits, enemy deaths, and misses. The enemy was hit if they were shot in a non-vital area such as hand, foot, or shoulder, while they were killed when shot in a vital area like head or chest. For the golf study, we calculated the percent of putts that ended up in the hole as the main performance metric.

### Algorithm development

EEG-derived engagement and workload, and ECG-derived heart rate metrics were combined together in order to analyze their common changes over time. To normalize the data for individual variability, all three metrics were self-normalized (i.e., z-scored) with respect to the training task in question. This accommodated for individual differences inherent in the physiological data. The feature vector was derived by applying a 3 s sliding window to each second of data (i.e., the window was shifted in 1 s increments). Thus, the feature vector comprised the three analyzed metrics (i.e., EEG-engagement, EEG-workload, and heart rate) over three consecutive seconds, summing up to 9 features.

These vectors were used for training an unsupervised self-organizing artificial NN (Kohonen, 1990) consisting of 20 nodes placed on a 1D grid (20 × 1), which is a discrete representation of the continuous input space with the preserved spatial properties of that space (i.e., topological relationships within the training set are maintained). In the NN, the nodes are represented with a weight vector (i.e., code vector) whose dimension is equal to the dimension of the input vectors and a neighborhood function that decays with distance and dictates the topology of the map. Training consists of the competitive learning process which develops the topology in the following manner. The feature vectors most similar to each other (based on the Euclidean distance) activate the same nodes, i.e., in the end similar nodes are closer to each other on the grid than the more dissimilar ones. For each input pattern, the node with the closest weight vector is declared the Best Matching Unit (BMU). The weights of the BMU and its neighbors are then adjusted toward the input vector. The neighborhood size is at first large during the ordering phase, and afterwards it is reduced in each iteration until only the BMU's weight starts to be updated during convergence phase when fine tuning of the NN weights is performed. After training, mapping is carried out by assigning each new feature vector to the NN node it activated. In our experiments, both training and mapping were performed in MATLAB® using Neural Nework Toolbox. The batch version of the learning algorithm was applied: instead of presenting only a single feature vector per iteration, all vectors are presented to the NN in each iteration and the weights change so that each node's new weight vector is the weighted average of the input vectors that activated that node. The weight vectors were initialized with the most significant principal components of the input space to start with a reasonable ordering. By distributing the weights in this manner and using the batch learning algorithm, the number of iterations needed to reach convergence was reduced, and we ran 200 iterations in our experiments.

The goal of the analysis was to find relevant patterns in the trained NN nodes' activations related to the skill training process. In our experiments, the NN model was trained on the combat marksmanship data that were also used for auto-validation as a proof of concept. The NN model was validated by utilizing 10-fold cross-validation. In the first nine cross-validation iterations, the data from five subjects were used for training and the rest of the data were used for testing. In the last round, the data from six subjects were employed for training and the rest of the data was employed for testing. By choosing the training folds in this manner, we evaluated person-independent modeling (i.e., the test data did not comprise the data from the subjects that were used for training) and every subject in the study was used only once in the training phase. The trained NN model was also tested on the golf study data to evaluate generalization capabilities of the model across different tasks. An extensive set of analyses was performed. First, we characterized the NN nodes by aggregating the feature vectors that activated the same node and analyzed the size of each node, i.e., how many times each node was activated. Second, the distributions of activated NN nodes during different relevant events and periods in both datasets were analyzed. The distributions were aggregated by counting all node activations whenever the event of interest appeared in the combat marksmanship dataset or during a certain period of interest in the golf dataset. The middle point of the 3 s long feature vector window was taken as a reference point, i.e., we analyzed EEG-engagement, EEG-workload, and heart rate metrics of the 1 s epoch when the event occurred, and their changes 1 s before and 1 s after the event. One-Way Multivariate Analysis of Variance (MANOVA) was applied to the estimated distributions to investigate if they are statistically different, i.e., if different neurophysiological patterns are associated with these events and periods of interest. Third, time series analysis of the adjacent NN node activations was performed by deriving a transition matrix that shows the overall number of the subsequent NN node activations for all possible pairs of the NN nodes. Fourth, based on the observed patterns in the transition matrix, we introduce a transition score that measures the size of temporal state changes as a sum of transitions between the subsequent activations of the NN nodes in the following manner. Let *t* be an overall number of activated nodes during the subject's skill training session (i.e., an overall number of feature vectors presented to the NN model), and let *i(j)* be the NN node activated during epoch *j* (*i* ∈ [1, *N*], *N* = 20, *j* ∈ [1, *t*]). We define transition score as:

$$Transition\;score=\;\sum_{j\;=\;2}^{t}abs(i\left(j\right)-i(j-1))$$

Correlation analysis was performed to investigate if these transition scores were related to the subject's performance scores in both studies. Lastly, these performance prediction results were compared to the linear regression model on the same set of variables used for training the NN model.

## Results

In this section, the following results are presented: the frequency of activated NN nodes and their patterns during the important events and periods of interest in both the combat marksmanship and golf training datasets, time series analysis of activated NN nodes throughout the entire training sessions obtained by calculating transition matrices, analysis of performance scores, correlations between transition scores and performance scores, and comparison with the linear regression model.

### Analysis of the NN nodes

**Figure 5:**
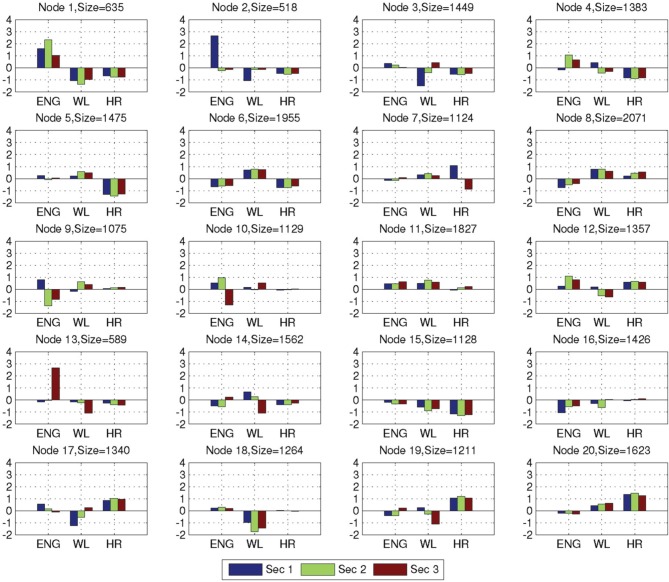
**Average variable values of EEG-engagement (ENG), EEG-workload (WL), and heart rate (HR) for the NN nodes, together with size of the nodes (i.e., the number of feature vectors that activated the corresponding node)**.

After training on the combat marksmanship dataset was completed, we grouped together and analyzed the feature vectors that activated the same NN nodes. Average EEG-engagement, EEG-workload, and heart rate values of the grouped feature vectors for each node and node sizes (i.e., a number of feature vectors that activated the nodes) are shown in Figure 5. The nodes 8, 6, and 11 were activated the most often (2071, 1955, and 1827, i.e., 7.9, 7.5, and 6.9% of the time), and they represent three distinct neurophysiological states in which there are no large changes in any of the three analyzed metrics. Node 8 represents a state of low EEG-engagement, as its z-scored value is negative (i.e., below the average value over the subject's entire training session) that is increasing over the observed 3 s. Heart rate is also slightly increasing and it is above the subject's average value, while EEG-workload is slightly decreased during the third second, but still above the average subject's value. Node 6 represents EEG-engagement and heart rate below the subject's average value, while EEG-workload is above the average value. Lastly, EEG-engagement and EEG-workload of node 11 are positive, while heart rate is around the subject's average value with a slight increase tendency. The least activated nodes were 2 and 13 — they were activated 518 and 589 times, i.e., 1.9 and 2.2% of the time, respectively. The main characteristic of these nodes is that they have a large decrease (node 2) or increase (node 13) in EEG-engagement and, at the same time, relatively large opposite change of EEG-workload. These two nodes were the least often activated nodes during the validation of the trained NN on the golf training data as well (0.8% node 2, and 0.9% node 13). The most often activated nodes for the golf dataset were also nodes 11 (10.4%) and 6 (7%), in addition to node 5 (7.5%) that has around average EEG-engagement, above average EEG-workload, and below average heart rate values.

**Figure 6:**
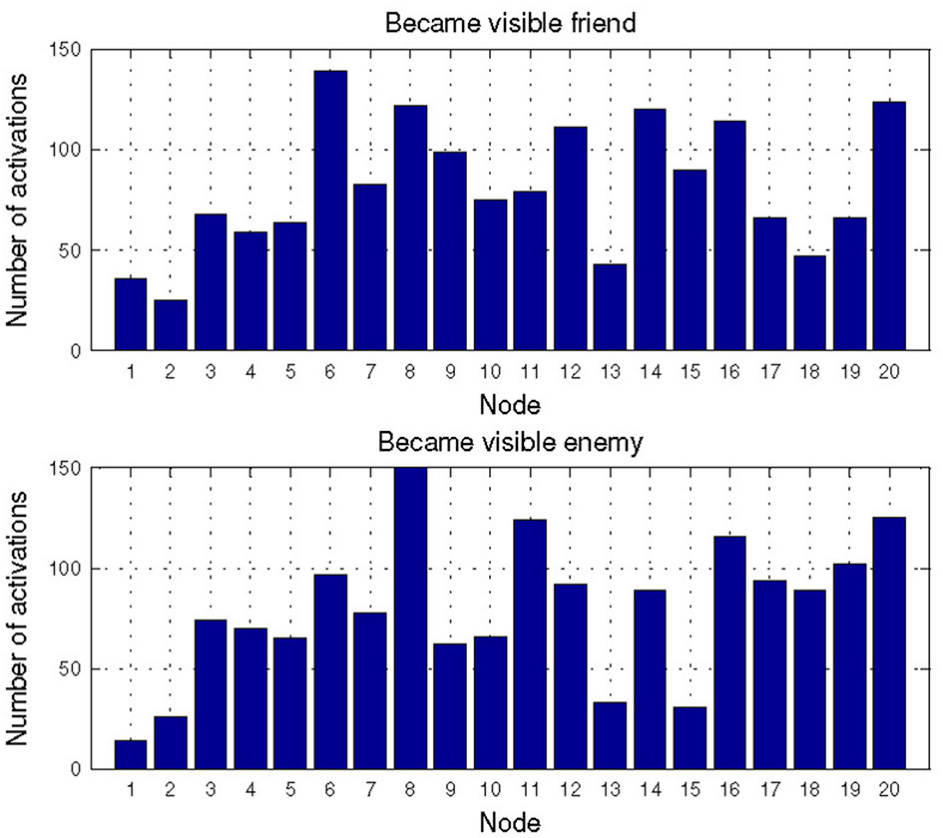
**Distribution of the activated NN nodes for the events: “Became visible friend” and “Became visible enemy” in the Combat marksmanship dataset**.

For the combat marksmanship dataset, we analyzed the changes in the subjects' EEG-engagement, EEG-workload, and heart rate when an enemy appeared and compared them to the typical subject's reactions to the friendly unit's appearance by examining distributions of activated NN nodes for these two events. In Figure 6 are shown distributions of the activated NN nodes for the two analyzed events (i.e., “Became visible friend” and “Became visible enemy”). MANOVA analysis showed statistically significant difference between these two events' NN node activations [*F*(20, 427) = 1.716, *p* = 0.028, effect size η^2^_partial_ = 0.07, observed power = 0.966]. When looking at differences in the individual node's activations, the largest difference was for nodes 15 and 11. Node 15 was activated more often as a response to the appearance of friendly units and node 11 was a frequent response to the enemy's appearance. Node 15 represents the relatively relaxed state when all three metrics (i.e., EEG-engagement, EEG-workload, and heart rate) are below their average values, while node 11 is a representative of above average EEG-engagement, EEG-workload, and heart rate values. Furthermore, there is a slight decrease of EEG-engagement and heart rate in node 15, during the third second, when friendly units appeared, while in node 11 an increase of EEG-engagement and heart rate is encountered during the third second, i.e., after enemies appeared.

**Figure 7:**
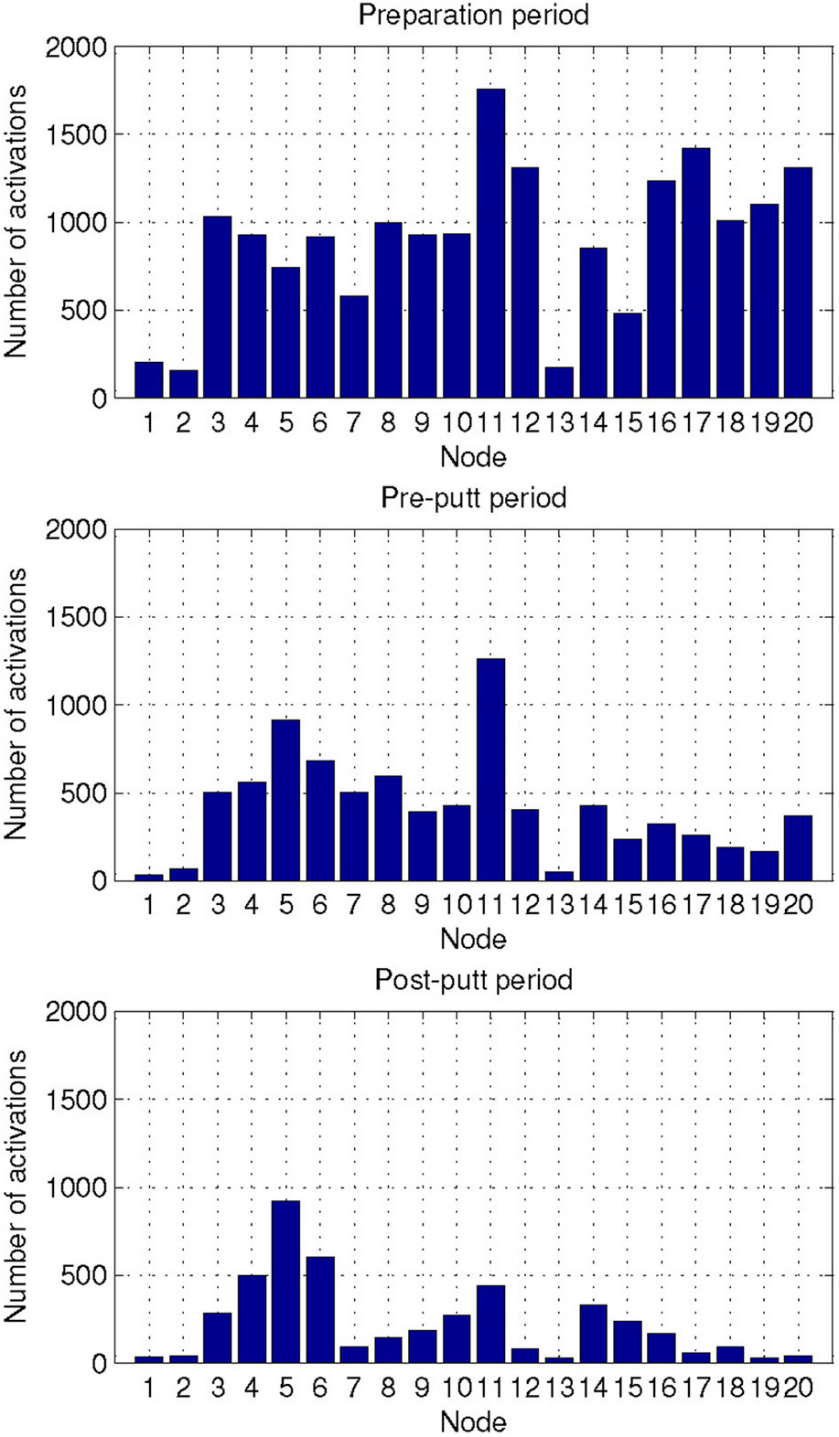
**Distribution of the activated NN nodes for the putt periods of the golf study: preparation, pre-putt, and post-putt**.

We also characterized the three periods of interest in the golf study: preparation period, pre-putt period, and post-putt period by their distribution of activated NN nodes. These results are shown in Figure 7. From the plots one can observe that the most frequent node during the pre-putt period was node 11, while node 5 was the most often activated during the post-putt period. Comparison of those 2 nodes' EEG-engagement, EEG-workload, and heart rate values (shown in Figure 5) indicated above average EEG-engagement, EEG-workload, and heart rate levels during the pre-putt period, while during the post-putt period EEG-engagement was back to its average value, heart rate decreased below average value, and EEG-workload was still above average but slightly decreased compared to the pre-putt period. Furthermore, we compared pre-putt and post-putt periods' node activation distributions for the hits and the misses, and MANOVA showed statistically significant differences between pre-putt and post-putt periods for these two types of putts [*F*(20, 2178) = 4.018, *p* < 0.001, effect size η^2^_partial_ = 0.036, observed power = 1 for pre-put period and *F*(20, 2128) = 1.743, *p* = 0.02, effect size η^2^_partial_ = 0.016, observed power = 0.974 for post-putt period].

### Transition matrices

**Figure 8:**
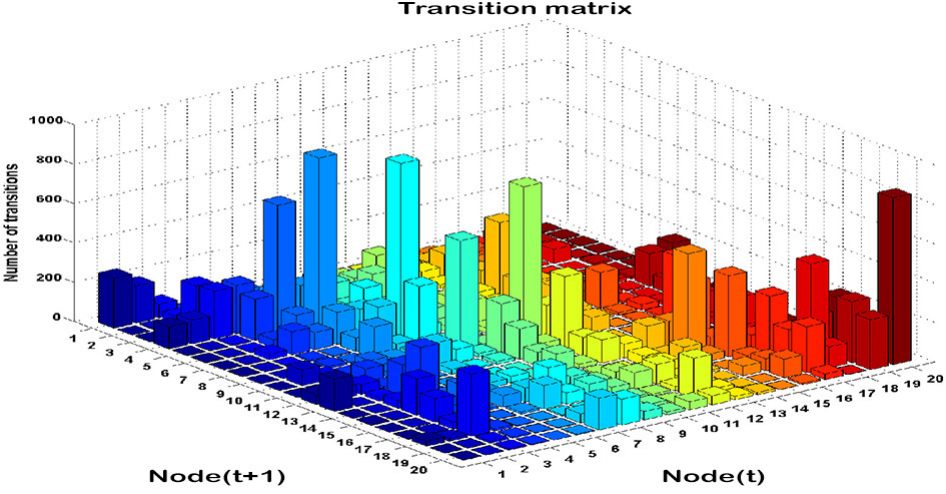
**Transition matrix during auto-validation on the combat marksmanship dataset: a number of subsequent transitions between each pair on the NN nodes**.

**Figure 9:**
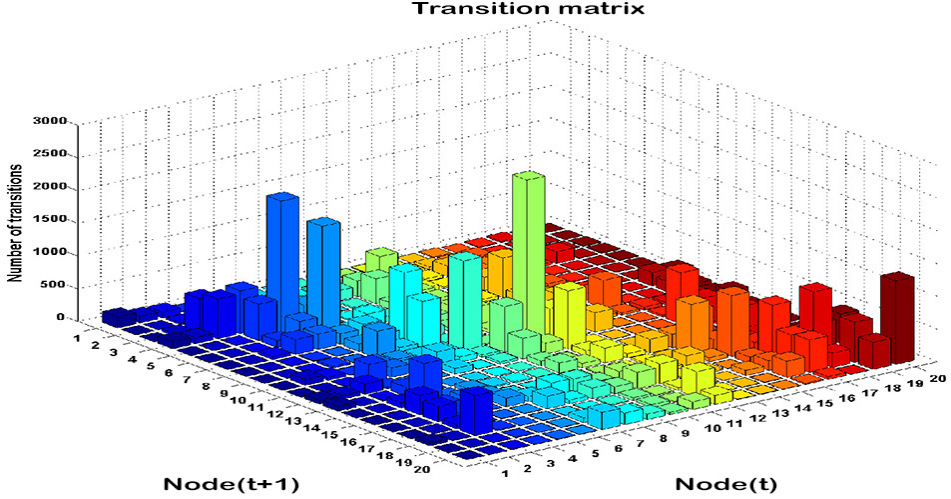
**Transition matrix during validation on the golf dataset: the number of subsequent transitions between each pair of the NN nodes**.

**Figure 10:**
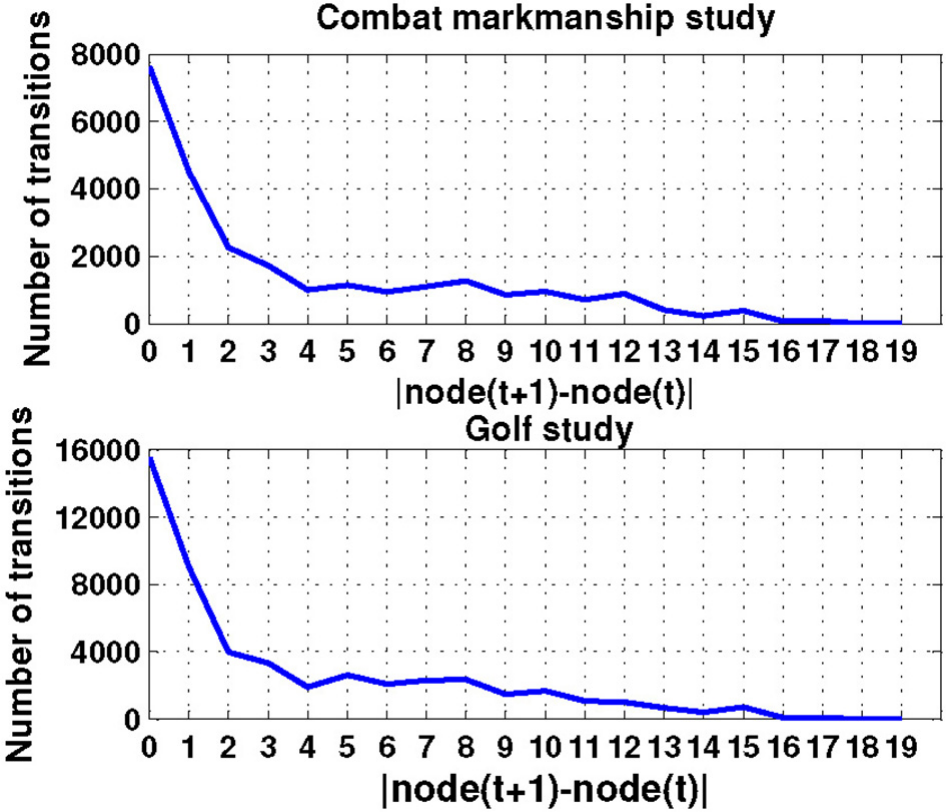
**The number of transitions aggregated over distance (i.e., position) of the nodes on the NN grid for both combat marksmanship and golf dataset**.

Time series analysis of the activated NN nodes was performed and transition matrices on the second-by-second basis were constructed. Transition matrices for both combat marksmanship and golf study are shown in Figures 8, 9, respectively. From the plots it can be observed that both transition matrices followed the same pattern of changes, i.e., most of the transitions were local, which was reflected in the transition matrix as movement around a diagonal. This is confirmed in Figure 10, which shows a number of transitions aggregated across distance between the nodes (based on their sole position on the NN grid, and not on Euclidean distances between them). From the plots it can be clearly seen that the number of transitions decreased almost exponentially with distance between the nodes in both datasets. This indicates that the analyzed physiological metrics were not changing dramatically over time. On the other hand, larger state changes were less dominant, however, after investigating their timestamps it turned out that they typically represented some significant events in the datasets that caused large increase or decrease of EEG-engagement, EEG-workload, or heart rate metrics. Consequently, more distant nodes of the trained NN model were activated, and in such cases an average nodes' distance on the NN grid was 4. In order to quantify this phenomenon, we calculated transition scores for each subject's skill training session and correlation analysis was performed to investigate if they were associated with the subject's performance.

### Analysis of performance scores

**Figure 11:**
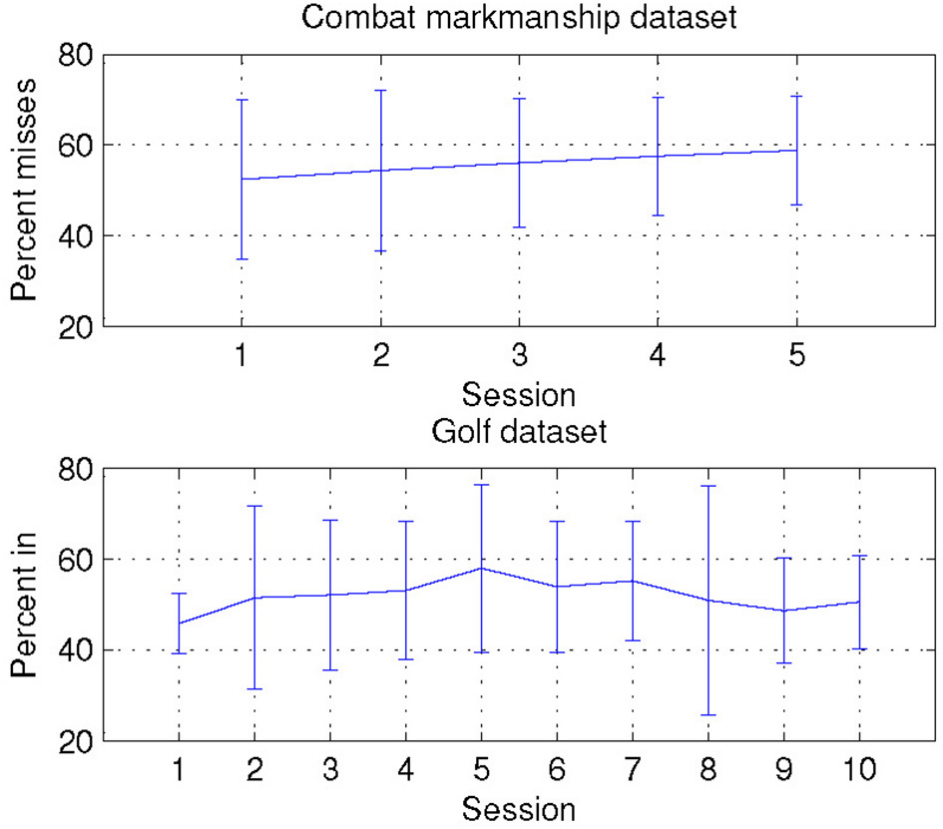
**Averaged subject's performance for both combat marksmanship and golf dataset**.

In the combat marksmanship dataset, even though the scenarios had varying number of enemies, there were no statistically significant differences in performance across different scenarios. The order of the scenarios was randomized across subjects, and there was a slight trend of decrease (about 5%) in performance in the later scenarios as shown in Figure 11, but it was not statistically significant (*p* = 0.45).

In the golf dataset, experienced players performed better on average (by 16%), but after ranking all subjects by the achieved performance, the set of the best performers comprised an equal split of novices and experienced players. Averaged subjects' performance for each session is shown in Figure 11, and there was an increase of 12% in performance during the first half of the sessions. Afterwards, performance started to decrease, but when comparing the first and the last session, there was an overall slight improvement in performance (by 4.8%).

### Correlation analysis

Correlations during auto-validation between transition scores and performance scores for combat marksmanship are shown in Table 1. Table 2 shows correlations during 10-fold cross-validation between transition scores and performance scores for combat marksmanship. In Table 3 are shown correlations between transition scores and performance scores for the golf study. In all three cases, the correlations were statistically significant based on the two-tailed *t*-test. Statistically significant positive correlations between transition scores and percent of enemy hits/enemy deaths for the combat marksmanship dataset, as well as between transition scores and percent of “in” putts for the golf study, indicate that the larger the transition score the better the performance. Negative correlations between transition scores and percent misses in the combat marksmanship dataset during both auto-validation (Table 1) and 10-fold cross-validation (Table 2) show a trend of lower transition scores for the worst performing subjects.

**Table 1:** **The Pearson's correlation coefficients during auto-validation between transition scores and performance scores for the combat marksmanship dataset**.

	***r***	***r*^2^**	***p*-value**
Percent enemy hits	0.149	0.022	0.027
Percent enemy deaths	0.187	0.035	0.007
Percent enemy misses	−0.21	0.044	0.003

**Table 2:** **The Pearson's correlation coefficients during 10-fold cross-validation between transition scores and performance scores for the combat marksmanship dataset**.

	***r***	***r*^2^**	***p*-value**
Percent enemy hits	0.148	0.022	0.027
Percent enemy deaths	0.173	0.029	0.012
Percent enemy misses	−0.203	0.041	0.004

**Table 3:** **The Pearson's correlation coefficients between transition scores and performance scores for the golf dataset**.

	***r***	***r*^2^**	***p*-value**
Percent “in” putts	0.119	0.014	0.048

### Comparison with the linear regression model

We built the linear regression model using the same set of variables as in the NN model to predict the performance scores in the combat marksmanship study. We tested the variables for multicollinearity by calculating tolerance and the corresponding variance inflation factor (VIF). The results are shown in Table 4. As tolerance > 0.2 (i.e., VIF < 5), one can conclude that multicollinearity was not present. Thus, the linear regression model was built without regularization. The regression results are shown in Table 5. One can observe that only the model that predicted the “percent enemy deaths” performance score was statistically significant (*F*-Test, *p*-value = 0.018). In that particular model, only 3 predictor variables were statistically significant: EEG-Engagement—sec 2 (*t*-test, *p*-value = 0.004), EEG-Workload—sec 1 (*t*-test, *p*-value = 0.05), and EEG-Workload—sec 3 (*t*-test, *p*-value = 0.05). When comparing the *r*^2^ scores in Table 1 (i.e., based on the NN model) and Table 5 (i.e., based on the linear model), one can conclude that the NN model outperformed the linear regression model, as it was able to predict the performance scores more accurately.

**Table 4:** **Multicollinearity test: VIF and tolerance of the EEG-engagement, EEG-workload, and heart rate variables**.

**Variable**	**Tolerance**	**VIF**
EEG-Engagement—sec 1	0.848	1.179
EEG-Engagement—sec 2	0.798	1.253
EEG-Engagement—sec 3	0.862	1.16
EEG-Workload—sec 1	0.585	1.708
EEG-Workload—sec 2	0.458	2.182
EEG-Workload—sec 3	0.593	1.685
Heart rate—sec 1	0.753	1.328
Heart rate—sec 2	0.642	1.558
Heart rate—sec 3	0.775	1.291

**Table 5:** **The linear regression model performance prediction results: coefficient of determination (*r*^2^), *p*-value for the *F*-test of the overall linear regression model, and the *p*-value for the *t*-test of the predictor variables**.

	**Percent enemy hits**	**Percent enemy deaths**	**Percent misses**
*r*^2^	0.001	0.004	0.002
*F*-test, *p*-value	0.71	0.018	0.18
EEG-Engagement—sec 1	0.36	0.34	0.34
EEG-Engagement—sec 2	0.33	0.004	0.02
EEG-Engagement—sec 3	0.25	0.27	0.29
EEG-Workload—sec 1	0.99	0.05	0.15
EEG-Workload—sec 2	0.24	0.07	0.48
EEG-Workload—sec 3	0.72	0.05	0.30
Heart rate—sec 1	0.16	0.34	0.25
Heart rate—sec 2	0.58	0.94	0.99
Heart rate—sec 3	0.49	0.63	0.99

## Discussion

In this paper, we explored a novel way of combining EEG-based metrics of engagement and workload, and an ECG-based heart rate metric to capture temporal cognitive state changes related to performance on two different skill training tasks, namely combat marksmanship and golf training. The main benefit of the algorithm is that it does not require prior labeling of the data as it employs the unsupervised self-organizing NN approach. The supervised learning approaches require large amounts of labeled data to train a classifier, but obtaining that large amount of labeled training data is a time-consuming and error-prone process that has limited the real-world applicability of many previous algorithms. Instead of utilizing low-level EEG features, we investigated the higher level EEG-based metrics of engagement and workload that contributed to the better generalizability of the approach across different tasks. The richness of the combat marksmanship dataset in the different EEG-engagement and EEG-workload levels allowed for successful training of the NN model. The model was able to cover all important patterns in cognitive state changes that were relevant for the golf task as well. In order to overcome individual differences inherent in the physiologically induced metrics, we normalized the data. Our algorithm was cross-validated in two different ways. First, person-independent training was performed by utilizing 10-fold cross-validation. Second, the NN model trained on the combat marksmanship data was tested on the golf dataset to evaluate generalization capabilities of the approach across different tasks. The temporal sequences of physiologically estimated cognitive state changes detected in the combat marksmanship dataset occurred in the golf dataset as well. The model was able to capture differences in the subject's psychophysiological states in response to different events such as appearance of the friendly and enemy units in the combat marksmanship scenarios or during different stages of golf putting. This could be also attributed to a difference in motor activity. We aimed to minimize that effect by excluding the epochs with detected EMG artifacts from the analysis. This demonstrated the richness of a model combining EEG-engagement, EEG-workload, and heart rate metrics that successfully provide a window on the internal mental state of the user. Analysis of the dynamic changes over time of these three metrics combined together contributes to a more in-depth understanding of different aspects and stages of skill training. For example, EEG-engagement plays an important role during gathering information necessary for performing a task, EEG-workload is essential in processing the gathered information and comparing it to internal mental models, while heart rate is an indicator of the subject's preparation to receive sensory inputs and reaction to the processed information.

Statistically significant correlations between the introduced NN nodes' transition scores and performance were found in both datasets, which supports our first two hypotheses that temporal sequences of EEG-based engagement and workload and ECG-based heart rate comprise patterns related to performance on the combat marksmanship dataset, and that the discovered patterns are good indicators of performance on the golf training task as well. This is in line with the findings of Stevens et al. (2013) that showed relationship between the fluctuations in the entropy of the activated NN nodes and the relevant events in the task that require significant cognitive re-organization. These large cognitive state transformations might reflect adaptation to changes in the task. In their study, decreases in entropy were also associated with periods of poorer task performance. Though preliminary, our results also suggest physiological signatures may distinguish elements of perception indicative of good performance. Unlike previous studies that analyzed the relationship between the performance scores on the combat marksmanship and golf training tasks (e.g., Crews and Lander, 1993; Haufler et al., 2000; Deeny et al., 2003; Babiloni et al., 2008), and low-level EEG features such as PSD bandwidths over different scalp regions, we focused on the higher level metrics such as EEG-engagement, EEG-workload, and heart rate. The discriminative patterns in these three metrics were general across the two analyzed tasks and could be included in future neurofeedback-based or other intelligent training systems to accelerate learning across different tasks. Beyond performance estimation and skill acquisition scenarios, the proposed unsupervised approach based on time series analysis holds potential to also enrich information systems with additional data on cognitive state changes of the user that could be employed, for example, for testing the effectiveness of different marketing advertisements, user's reactions to different movie storyline endings, or even to political speeches. Due to the general nature of the developed algorithm, different NN nodes would be more active under diverse application scenarios, and the speed of the feature vector changes specific to different applications could be captured.

Even though the average ages of the subjects in these two datasets are different (27.4 ± 6.7 in the combat marksmanship dataset vs. 33.9 ± 11.1 in the golf dataset) and physiological signals are age dependent, the transfer of learning over these two datasets was successful, presumably due to the normalization of data. Furthermore, the EEG-based engagement metric is derived from the model individualized for each subject based on the EEG data during the baseline VPVT, APVT, and 3CVT tasks. Utilization of individualized metrics such as EEG-based engagement further facilitates knowledge transfer between different tasks. Although the algorithm proposed in our work is built upon the approach of Stevens et al. (2009, 2013) we extended it in a number of ways. First, we did not analyze the three metrics separately, but we combined EEG-engagement, EEG-workload, and heart rate into a single NN to capture synchronous changes of the metrics. Second, we did not apply a quantization into the upper, middle, and lower quartile, rather, we analyzed the metrics on a fine-grained continuous scale to be able to capture subtle cognitive changes during the tasks. Third, we incorporated temporal modeling in the NN itself by including 3 consecutive seconds of data into a feature vector. Thus, already the NN nodes were, to some extent, able to capture relevant temporal patterns in the data. Further investigations are needed to estimate the optimal window size, but increasing it from 1 to 3 s allowed for better temporal modeling and interpretation of the NN nodes. Fourth, we reduced the number of the NN nodes from 25 [that was used in Stevens et al. (2009, 2013)] to 20, which proved to be sufficient for our model and at the same time, reduced the time required to train the model, resulting in a more efficient algorithm. Fifth, we introduced a simple, yet effective NN state transition score that was correlated with performance scores in both analyzed datasets. Lastly, Stevens et al. (2009, 2013) focused on the analysis of team settings, which present a number of confounding factors, but the goal of this study was to analyze the individual changes in cognitive states and to eventually reveal the patterns relevant for characterizing performance on different tasks. We will also investigate the potential of better feature interactions coverage in the two-dimensional NN grid.

The current study sought to develop a method for capturing cognitive state changes that could be integrated in a number of applications, but this is only the first step in developing a performance estimation system that could be implemented in real-time and utilized in real-world settings. In order to apply the proposed approach in real-time, after the NN model is trained, one would need to collect the appropriate baseline data for new users to allow for individual data normalization (i.e., z-scoring utilized in our algorithm). Another option would be to compute mean and standard deviation of the subject's EEG-engagement, EEG-workload, and heart rate metrics (that are necessary for z-scoring) adaptively online during the task itself. Thus, the algorithm could be relatively easily implemented in real-time. The main limitation is the 1 s delay required to capture the third second of data in our feature vector. In most of the envisioned potential applications of the proposed approach, this delay would be acceptable.

Furthermore, we analyzed different performance scores, such as percent of enemy hits and enemy deaths in the combat marksmanship study, to evaluate if we can estimate subtle differences in performance by capturing neurophysiological differences in the subjects' reactions to relatively similar events in this very rich dataset. However, larger studies are necessary in order to further refine the NN model to be able to also capture neurophysiological correlates of performance improvement. In the combat marksmanship study there was no clear overall improvement in performance throughout the scenarios, while in the golf study, performance was slightly improved at the end of the study compared to the initial performance, but there was a drop in performance seen in the second half of the golf sessions. As the golf study sessions lasted longer than the combat marksmanship sessions, the decrease in performance in the golf study was presumably due to a loss in concentration as the study progressed. Thus, a larger number of skill training sessions across different days is needed. We utilized simulated training scenarios in these initial studies to minimize the noise that could be introduced by environmental variables in the real-world settings, however, the training tasks were made as realistic as possible by working closely together with established military and golf training domain experts. We plan to extend the combat marksmanship study and include professional soldiers in order to obtain more realistic data, and compare their physiological profiles with the novices' profiles. Further validation on the additional tasks, such as driving, is also planned. Nonetheless, the statistically significant correlations between the NN-based transition scores and performance are a promising first step toward the final goal of performance prediction that could be further used across a wide range of real-world application scenarios to improve, accelerate, and increase efficiency of the skill learning process by identifying an adaptive, intelligent, and multimodal neurofeedback based on the relevant neurophysiological patterns discovered in our datasets.

### Conflict of interest statement

Authors Stikic, Berka, Levendowski, Rubio, Tan, and Korszen are paid salaries and/or shareholders of Advanced Brain Monitoring, Inc. The authors declare that the research was conducted in the absence of any commercial or financial relationships that could be construed as a potential conflict of interest.
